# Combined Cutaneous Sarcoma: Pleomorphic Liposarcoma and Conventional Osteosarcoma—The So-Called Malignant Mesenchymoma

**DOI:** 10.1155/2012/749898

**Published:** 2012-10-02

**Authors:** David Parada, Karla B. Peña

**Affiliations:** ^1^Servei de Patologia, Hospital Universitari Sant Joan de Reus, 43204 Tarragona, Spain; ^2^Institut d'Investigació Sanitaria Pere Virgili (IISPV), Universitat Rovira i Virgili, Reus, Tarragona, Spain

## Abstract

Malignant mesenchymoma is combined soft tissue tumors of mesenchymal origin. Cutaneous combined sarcomas are exceedingly rare. We report the case of an 81-year-old woman who presented a left cutaneous mass. She underwent a wide local excision. Histopathological and immunohistochemical studies were consistent with the diagnosis of combined pleomorphic liposarcoma and conventional osteosarcoma (malignant mesenchymoma). Although it is extremely rare, this case suggests that combined sarcoma should be considered in the differential diagnosis of undifferentiated pleomorphic neoplasms.

## 1. Introduction

Cutaneous soft tissue sarcomas (CSTSs) represent less than 1% of malignant tumors [1]. These heterogeneous mesenchymal neoplasms are relatively uncommon and compromise a broad range of differentiation and are classified histologically according to the mature tissue they resemble, many of which have more than one subtype [1, 2]. The term malignant mesenchymoma (MM) has been applied to sarcoma that exhibits two or more lines of specialized differentiation [3]. MM is an uncommon soft tissue tumors, first described by Stout in 1948 [4]. These tumors predominantly involve retroperitoneum and chest wall [5]. However, another localization has been described such as heart, lung, pleura, and thyroid [6–9]. 

In the WHO classification of skin tumors [2] MM, liposarcoma, or osteosarcoma were not classified as an entity among the tumors of the skin or dermal structures. To our knowledge, a sarcoma, like our case where two lines of specialized differentiation were present in cutaneous region, has not been described to date. We discuss the differential diagnosis of combined sarcoma in this location.

## 2. Case Report

**Figure 1:**
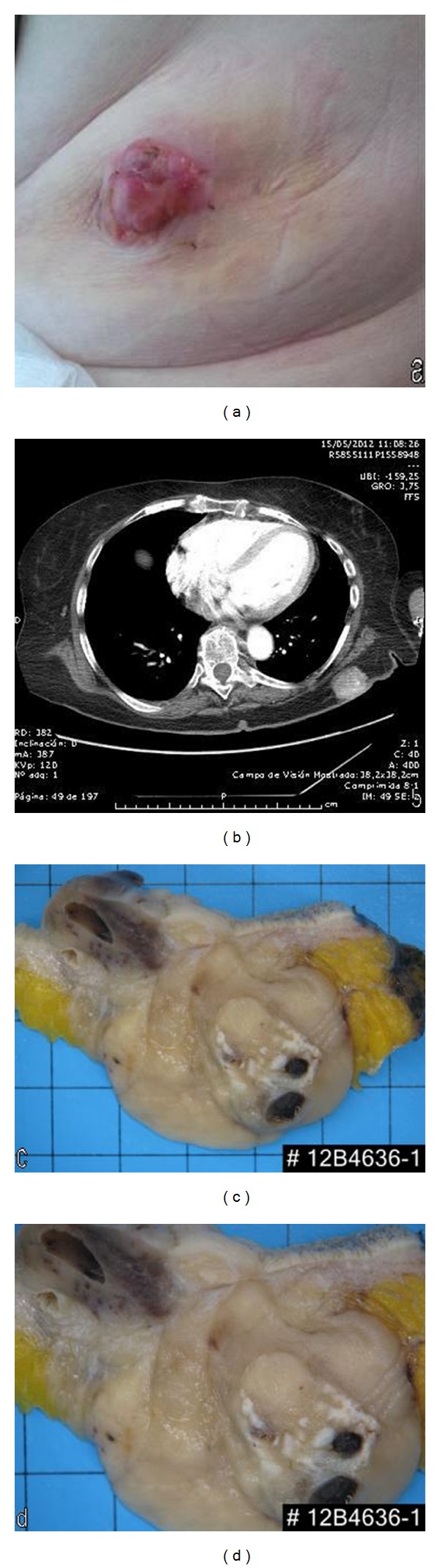
(a) Skin lesion showing an irregular lobular shape pink tumor. (b) Cranial tomography showing a tumoral lesion in the left skin region. Isolated calcifications are present. (c), (d) Surgical resection showing a solid multinodular calcified tumoral lesion, (note that no relation with skin is evident).

An 81-year-old woman visited the outpatient clinic with a several years history of a painless dermal mass in her skin left lumbar region. She had a previous history of hypertension and cholecystectomy. On physical examination, a left skin lumbar lesion was found (45 mm diameter) (Figure 1(a)). Thoracic computed tomography (CT) showed a left infrascapular mass with epidermal and dermal affectation (Figure 1(b)). A punch skin biopsy was performed. Preliminary report was undifferentiated neoplasia consisting with sarcomatoid squamous carcinoma. After review and histologic diagnosis the patient underwent wide local excision.

### 2.1. Pathology

#### 2.1.1. Macroscopic Findings

A surgical specimen revealed a 45 × 40 mm solid white, calcified mass dermal, well defined. Macroscopically, no skin relation was seen. The mass showed focal necrosis (Figures 1(c) and 1(d)).

#### 2.1.2. Microscopic Findings

**Figure 2:**
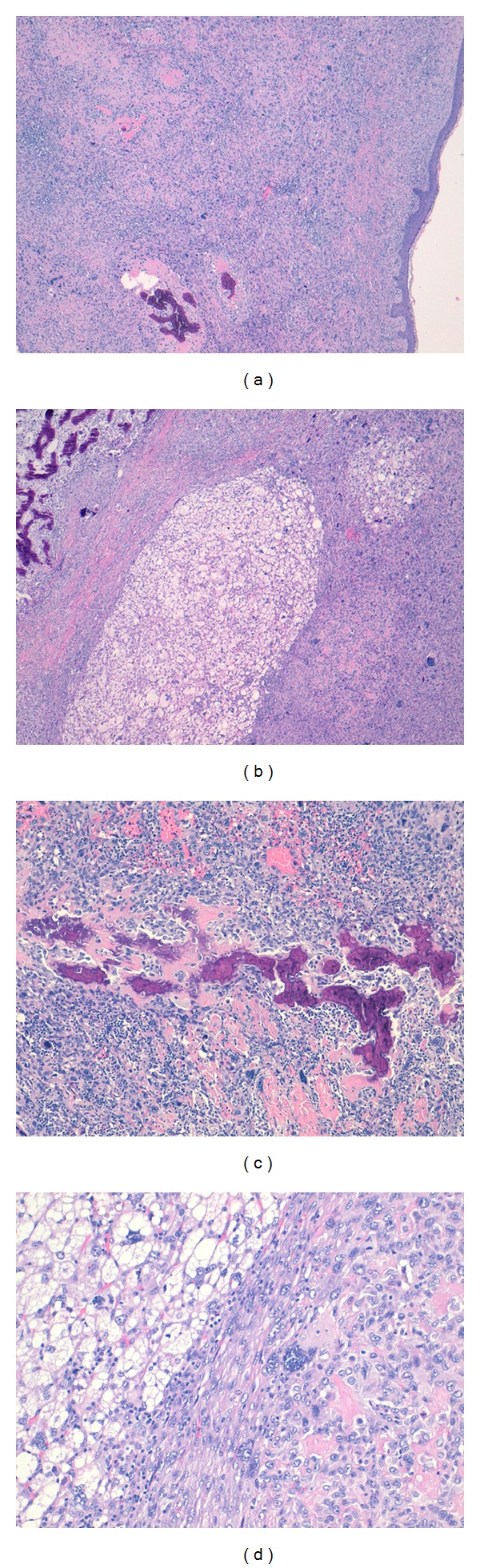
Histopathologic findings. (a) Sarcoma showing no relation with epidermis (noted a focus of osteosarcoma is present). (b) Combined sarcoma showing liposarcoma and osteosarcoma zones. (c) Typical calcified osteoid in conventional osteosarcoma. (d) Atypical pleomorphic lypoblasts adjacent to osteoid formation.

The tumor showed a proliferation of two different histopathological patterns. The most prominent histopathological pattern consisted of neoplastic cells with marked nuclear pleomorphism, conspicuous chromatin abnormalities, prominent nucleoli, and many mitotic figures, some of which are atypical. A dense, pink, amorphous intercellular material (osteoid) was present. The osteoid showed variable patterns and degrees of mineralization with focus of characteristic filigreed pattern. Focally, the tumor showed a spindle and pleomorphic multinucleated cells, resembling a malignant fibrous histiocytoma. Focally, another histopathological pattern was present in the tumor, characterized by a varying number of pleomorphic, multivacuolated lipoblast and admixed with spindle-shaped tumor cells with multinucleated giant cells (see Figure 2).

Immunohistochemical studies showed positivity of the neoplastic cells for vimentin and p63. S-100 protein showed a focal positivity in lipoblast. The tumor cells were negative for cytokeratin AE1/AE3, CAM5.2, desmin, smooth muscle actin, melan-A, and HMB45. 

The final diagnosis was combined cutaneous pleomorphic high-grade sarcoma: pleomorphic liposarcoma and conventional osteosarcoma (malignant mesenchymoma).

## 3. Discussion

Cutaneous soft tissue sarcomas are a heterogeneous group of mesenchymal neoplasms. Kaposi sarcoma is among the most common CSTS, followed by dermatofibrosarcoma protuberans, malignant fibrous histiocytoma, leiomyosarcoma, and angiosarcoma [1]. Other specified and no specified CSTS represent approximately 1.8% [1]. Malignant mesenchymoma (MM) is a sarcoma that exhibits two or more lines of specialized differentiation [1]. Our case showed definite pleomorphic liposarcoma and conventional osteosarcoma, so the diagnosis of MM was authentic according to the criteria presented in the WHO classification of tumors of soft tissue and bone. We present a case of MM, consisting of pleomorphic liposarcoma and conventional osteosarcoma in the cutaneous region that was diagnosed using a combination of morphologic and immunophenotypic characterizations. To the best of our knowledge, this is the first case report to describe this unusual combination presenting in the cutaneous region.

It has become apparent that MM does not make up a clinicopathological entity, and those potential candidates for the designation can be more appropriately classified in other ways [1]. Actually, sarcomas displaying two or more lines of differentiation are best diagnosed by identifying the lines of differentiation, their approximate amounts, and the grade of the most aggressive component [10]. If we address this change of concept, purely dermal liposarcoma is exceedingly rare [11–15]. In pleomorphic liposarcoma, nonlipogenic described areas are characterized by malignant fibrous histiocytoma-like, round cell liposarcoma-like, epithelioid/carcinoma-like features and/or cartilaginous, osseous, smooth muscle or skeletal muscle elements [16]. Our case represents an unusual combination of two exceedingly rare combined sarcomas in the cutaneous region.

The differential diagnosis of sarcoma, in the cutaneous region, includes atypical fibroxantoma, sarcomatoid carcinoma, melanoma, malignant fibrous histiocytoma, fibrosarcoma, leiomyosarcoma, and rhabdomyosarcoma. The spindled variant of atypical fibroxanthoma (AFX) shows similar histopathologic findings, and most reliable markers to differentiate AFX from another sarcomas are CD10 and S-100. AFX is usually positive for CD10 and S-100 is only expressed in dendritic cells (Langerhans cells), which are nonneoplastic cells commonly present within AFX [17]. Sarcomatoid carcinoma shows pleomorphic areas and cytokeratin, p63 expression. The last immunoprofile should be taken into consideration carefully, because liposarcoma and osteosarcoma can showed positivity to this antibodies, and erroneous conclusive diagnosis can be made in small biopsies. Another important differential diagnoses are melanoma and malignant peripheral nerve sheath tumor (MPNST), because these lesions are S-100 positive. However, HMB45 and Melan A are usually positive in melanoma and negative for liposarcoma or osteosarcoma. Smooth muscle actin, desmin, and myoglobin D1 can be used to establish the diagnoses of leiomyosarcoma, rhabdomyosarcoma.

In summary, we report a case of an 81-year-old woman presenting with tumor in the cutaneous region. Combined cutaneous sarcoma was diagnosed by integrating histological and immunophenotypic studies. It is a rare tumor in an uncommon site, and in this paper we review the entity, as well as the main histological differential diagnosis.
